# Are the anomalous vertebral arteries more hypoplastic?: retrospective linear mixed model approach

**DOI:** 10.1186/s12883-017-0951-x

**Published:** 2017-08-29

**Authors:** Chulho Kim, Jong-Hee Sohn, Hui-Chul Choi

**Affiliations:** Department of Neurology, Chuncheon Sacred Heart Hospital, Chuncheon-si, Gangwon-do 200-704 Republic of Korea

**Keywords:** Vertebral artery, Abnormalities, Computed tomography angiography, Magnetic resonance angiography

## Abstract

**Background:**

Small or hypoplastic vertebral artery (VA) is one of the risk factor for posterior circulation stroke. We assess whether various types of VA anomaly contribute to its diameter.

**Methods:**

We screened 238 patients who underwent neck CT and MR angiography within 1 month. A V1 anomaly was defined as the abnormal origin of the VA on a three-dimensional MR angiography and a V2 anomaly was defined as the VA not passing through the 6th cervical transverse foramen (TF) on an axial CT image. A linear mixed model was used to evaluate the determinants of VA size.

**Results:**

Among participants, 24 (10.1%) subjects exhibited an anomalous VA and, of the 476 VAs examined, 11 (2.3%) had an aortic origin and 27 (5.7%) had an abnormal entrance into the C6 TF. Presence of the V1 anomaly was positively associated with the V2 anomaly (*P* for chi-square < 0.001) and a linear mixed model revealed that being male (0.2 mm larger, *P* = 0.015), having a right VA anomaly (0.3 mm smaller, *P* < 0.001), having a V1 anomaly (0.9 mm smaller, *P* < 0.001), and having a V2 anomaly (0.7 mm smaller, *P* < 0.001) were significant predictor of VA diameter.

**Conclusion:**

The diameters of VAs with an anomalous aortic origin or an abnormal entrance of the TF were significantly smaller than those of normal VAs. These findings suggest that anomalies of the VA detected in 3-dimensional CTA or MRA may be clues for vertebral artery hypoplasia.

**Electronic supplementary material:**

The online version of this article (10.1186/s12883-017-0951-x) contains supplementary material, which is available to authorized users.

## Background

Vertebrobasilar atherosclerosis is a common etiology of posterior circulation strokes (PCS), which account for 20% of all ischemic strokes [1, 2]. Although there is a high prevalence of vertebral artery hypoplasia (VAH) among the normal population [3], VAH also greatly contributes to an increased risk of PCS [3–5]. However, the rates of VAH reported in the literature are quite variable because there is no standard definition for this disorder as of yet [4–8].

During embryological development, the vertebrobasilar artery forms from paired neural arteries that receive their blood supply from carotid-vertebrobasilar anastomoses such as the trigeminal, otic, hypoglossal, and proatlantal arteries [9, 10]. At the same time, the longitudinal anastomoses of the cervical intersegmental arteries contribute to the development of the vertebral artery (VA) which, in turn, is connected to the subclavian artery [11, 12]. The exact nature and precise underlying mechanisms of persistent carotid-vertebrobasilar anastomoses have yet to be fully characterized, but some researchers have suggested that these anastomoses remain unchanged to compensate for the delayed development of the vertebrobasilar artery [12, 13]. In other words, VAH is the product of the delayed development of the vertebrobasilar artery and might be associated with anomalies in posterior circulation.

**Fig. 1:**
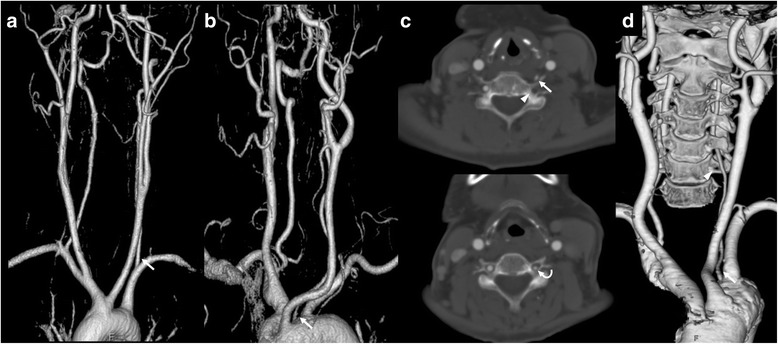
Left VAs (*arrows*) originating from (**a**) the subclavian artery and (**b**) the aorta on a three-dimensional MR angiography. Axial source images (window level = 400 Hounsfield Units, width = 1400 Hounsfield Units) of the CT angiography showing that the left VA (**c**, *arrow*) did not penetrate the C6 transverse foramen (*arrow head*) but that it passed through the C5 transverse foramen (**c**, *curved arrow*). Three-dimensional reconstructed CT image (**d**) showing a left VA (*arrow*) with an aortic origin and an empty left C6 transverse foramen (*arrow head*)

It is important to distinguish between acquired narrowing and hypoplasia of the VA because each of these has different atherosclerotic burden. Even in the case of digital subtraction angiography, which is the gold standard method for assessing cerebral arterial pathology, it is difficult to distinguish between atherosclerotic narrowing and hypoplasia because it only evaluates internal lumen of the blood vessel. The present study hypothesized that certain vascular anomalies of the VA would be associated with a small or hypoplastic VA. For example, the VA usually arises from the subclavian artery (Fig. 1a) but 2-5% of all VAs originate directly from the aorta [14–17]. Additionally, the VA typically penetrates the transverse foramen (TF) of sixth cervical vertebra (C6) but approximately 5-10% of VAs do not pass through this area (Fig. 1b) [17–19]. These anomalies are relatively common, which account for ~5% of incidence in general [15, 19]. Thus, the present study aimed to determine whether an anomalous origin of the VA and/or its abnormal penetration of TF other than C6 would contribute to the VA diameter in hospital-based populations.

## Methods

### Participants

The present study was a single-center retrospective observational investigation. Inclusion criteria for this study were a subject who visited our neurology clinic and underwent computed tomography (CT) and magnetic resonance (MR) angiography from January 2012 to December 2015. Additionally, the CT and MR images of each subjects were taken within a 1-month interval (*n* = 279 subjects). We excluded subjects who had motion artifacts on the CT or MR images (*n* = 12), insufficient arterial enhancement on the CT image (*n* = 23), and missing clinical information (*n* = 4). Additionally, patients who had profound vertebral artery tortuosity (*n* = 2) were excluded because vascular tortuosity could affect the radiologic parameter measurement of the VA. Our study is retrospective in nature, therefore, written informed consent was not obtained. However, the design of this study was approved by our Institutional Review Board/Ethics Committee.

### Definitions of anatomical variations

The VA usually originates from the subclavian artery and can be divided into four anatomical parts along the course of craniocervical area [20]. The V1 segment arises from the subclavian artery and extends to the proximal portion of the C6 TF, the V2 (transforaminal) segment extends from the C6 to the C1 TF, the V3 segment extends from the C1 to the dura, and the V4 segment extends from the dura to the vertebrobasilar junction. In the present study, the anomalous origin of the VA was referred to as a V1 anomaly and consisted of the VA directly originating from the aorta, as shown on a three-dimensional MRA (Fig. 1b). A V2 anomaly was defined as a VA that did not pass through the C6 TF (Fig. 1c and d) on an axial source CTA. A window level and a width setting of 400 and 1400 Hounsfield Units, respectively, were selected to optimally visualize the V2 segment anomalies on the CT images.

### Measurements of the radiologic parameters

A 64-section multidetector CT scanner (Somatom Sensation 64, Siemens AG Medical Solutions; Erlangen, Germany) was used to identity V2 segment anomalies using the following settings: 120 kVp, 100 mA, slice thickness of 1.5 mm, and 512 × 512 matrix. All MR imaging was performed on a 1.5 T MRI system (Intera, Philips Medical Systems; Best, the Netherlands) with the following settings: slice thickness of 1.3 mm, TR = 3.5, TE = 1.2 ms, field of view = 70 × 70, flip angle = 40°, and acquisition matrix = 256 × 256. Additionally, real-time MRA was conducted to determine whether there was an anomalous origin of the VA (aortic origin) by administering a dynamic three-dimensional gadolinium injection at a rate of 4 ml/s. The identification of VA anomalies and the measurement of the VA diameters were performed in a pi-view workstation (Infinitt; Seoul, Korea) by two blinded investigators (C Kim and H-C Choi) Because there were many cases of stenotic narrowing and vascular tortuosity, which could augment stenotic degree of the VA, the VA diameter was measured at 1 cm above its origin on the MRA, and defined as the longest diameter perpendicular to the direction of the VA.

### Statistical analysis

These data were freely available from the online (Additional file 1). The baseline characteristics of the patients were summarized using proportions for categorical variables and means ± standard deviations for numerical data. Descriptive comparisons between the anomalous VA and normal VA groups were assessed using the chi-square test or the Mann-Whitney U test for categorical variables and Student’s t-test or paired t-test for continuous variables, as appropriate. The inter-rater reliability of the two blind investigators in terms of the VA diameter measurements was analyzed using the intraclass correlation value and the diameters of the VA were entered into dependent variable. Linear mixed models used to investigate the association of the diameters of the VA with anatomical and demographic characteristics, modeled as fixed effects (including age, sex [female vs. male], the side of the VA (right-sided vs. left-sided; within-subject variable), V1 anomaly [yes vs. no], and V2 anomaly [yes vs. no]). The compound symmetry of the variance-covariance matrix was used for the repeated measure analysis because the variance and correlation (covariance) between the right and the left VAs were constant.

## Results

### Patient characteristics

Of the 238 patients, the mean age (± standard deviation) of the population was 65.9 (± 12.1) years and the proportion of females was 41.6% (99 of 238 patients). Nearly half of the patients (45%) had experienced an ischemic stroke followed by headaches and/or dizziness with intracerebral hemorrhage (Table 1). Twenty four subjects (10.1%) exhibited either a V1 (aortic origin) or V2 (abnormal penetration of the TF) anomaly. Patients with an anomalous VA were significantly younger than patients who had a normal VA (*P* < 0.030) but there were no significant differences in terms of gender or comorbid conditions between these two groups.

**Table 1:** Clinical characteristics of the anomalous and non-anomalous VA groups

	Total (*n* = 238)	Anomalous (*n* = 24)	Non-anomalous (*n* = 214)	*P*
Female (%)	101 (42.4%)	12 (50%)	89 (41.6%)	0.429
Age, years (mean ± SD)	65.9 (±12.1)	60.8 (±12.1)	66.5 (±11.9)	0.030
Comorbidities				0.244^a^
Ischemic stroke	107 (45.0%)	8 (33.3%)	99 (46.3%)	
Headache/Dizziness	42 (17.7%)	8 (33.3%)	34 (15.9%)	
Intracerebral hemorrhage	31 (13.0%)	4 (16.7%)	27 (12.6%)	
Neoplasm	30 (12.6%)	2 (8.3%)	28 (13.1%)	
Others	28 (11.7%)	2 (8.3%)	26 (12.1%)	
V1 anomaly, number (%)^b^	11 (2.3%)	11 (22.9%)	-	-
V2 anomaly, number (%)^b^	27 (5.7%)	27 (56.3%)	-	-

*SD* Standard deviation, V1 anomaly = vertebral artery with an aortic origin; V2 anomaly = vertebral artery with an abnormal entrance to the transverse foramen

^a^Pearson’s chi-square test

^b^proportions in a total of 476 vertebral arteries

### Anomalies of the VA

Of the 476 VAs (from 238 patients) that were evaluated in the present study, 11 (2.3%) had an anomalous origin directly from the aorta and 27 (5.7%) did not have a normal penetration through the C6 TF (Table 2). Of the 11 VAs with an anomalous origin, nine (81.8%) had an aortic origin and were preferentially located on the left side. Of the 27 VAs with an abnormal entrance into the C6 TF, there were slightly more on the right side than on the left side (15 vs. 12, respectively) and the most frequent anomaly was a penetration of the C5 TF in the V2 segment (*n* = 23, 85.2%) followed by a penetration of the C4 (*n* = 3, 11.1%) and C3 (*n* = 1, 3.7%) TF. Additionally, eight of the VAs exhibited anomalies that included both an aortic origin and an abnormal entrance into the C6 TF. An anomalous origin of the VA was associated with an abnormal entrance into the C6 TF (chi-square test, *P* < 0.001; Table 3).

**Table 2:** Incidence of anomalies of the VA according to laterality of the VA

	Right	Left	Total
V1 segment			
Normal	236	229	465
Anomalous	2	9	11
V2 segment			
Normal	223	226	449
Anomalous	15	12	27
C5^a^	14	9	2
C4^a^	1	2	3
C3^a^	0	1	1

Data are presented as number

^a^VA Entrance to the transverse foramen at each level. For example, C5 represents VAs not passing through C6 but C5 transverse foramen

**Table 3:** Associations between an abnormal origin and an abnormal penetration of the VA into the transforamen

		MRA		Total
		V2 anomaly (+)	V2 anomaly (−)	
CTA	V1 anomaly (+)	8 (1.7%)	3 (0.6%)	11 (2.3%)
	V1 anomaly (−)	19 (4.0%)	446 (93.7%)	465 (97.7%)
Total		27 (5.7%)	449 (94.3%)	476 (100%)

*P* for chi-square < 0.001

*V1* VA with aortic origin in V1 segment, *V2* VA with an abnormal entrance into the C6 transverse foramen

### Relationships between VA diameter and anomalies of the VA

**Fig. 2:**
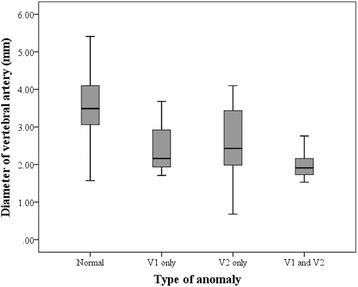
*Box plots* showing the median and interquartile range values for the VA diameters. V1: VA with an aortic origin anomaly; V2: VA with an abnormal entrance into the transverse foramen

The inter-rater correlation coefficient for the measurement of the VA diameter was 0.91 (95% confidence interval [CI]: 0.85-0.94, *P* < 0.001). The diameters of the VAs with either type of anomaly were significantly smaller than those with a normal morphology (Fig. 2). In the case of a VA with an anomalous origin or an abnormal foraminal entrance, the diameters of the left VAs were significantly smaller than those of the right side. Additionally, eight of the VAs exhibited a combined aortic origin and abnormal foraminal entrance; these were exclusively located on left side and had the smallest diameter of all the measured VAs.

Table 4 illustrates the findings of the linear mixed model analysis regarding the diameters of the VAs. Age did not significantly predict VA diameter but the diameters of the VAs in males were 0.2 mm larger than those in females (*P* = 0.015) and the diameters of the right VAs were 0.3 mm smaller than those of the left VAs (*P* < 0.001). Moreover, the diameters of the VAs with either type of anomaly (V1 anomaly, 0.9 mm smaller, *P* < 0.001; V2 anomaly, 0.7 mm smaller, *P* < 0.001) were considerably smaller than those of normal VAs, respectively. The (anomalous origin)*(abnormal entrance) interaction term was also included in the present linear mixed model but it was not significant. Therefore, this term was removed from the final analysis.

**Table 4:** A linear mixed model analysis revealed associations between VA diameter and the clinico-anatomical characteristics of the patients

Predictor	Β^a^ (mm)	95% CI	*P*
Age, years	0.0004	−0.005 – 0.006	0.881
Female^b^	reference		
Male	0.174	0.033 – 0.315	0.015
Left VA^b^	reference		
Right VA	−0.351	−0.484 – −0.217	< 0.001
V1 normal^b^	reference		
V1 anomaly	−0.916	−1.420 – −0.412	< 0.001
V2 normal^b^	reference		
V2 anomaly	−0.783	−1.112 – −0.454	< 0.001

^a^Reference

^b^Estimates of fixed effects (VA diameter, mm)

A power analysis with a 0.05 alpha error was performed using the results of the linear mixed model to evaluate differences in the diameters of the VAs between the anomalous V2 and normal V2 patients; the diameters of the anomalous VAs and the normal VAs were 2.4 and 3.48 mm, respectively. The actual power for the repeated measures analysis using G*Power 3.1 (Franz Faul, Christian-Albrechts-Universität Kiel; Kiel, Germany) was 0.93.

## Discussion

In the present retrospective case-control study, the diameters of the VAs of patients with two types of VA anomaly were assessed from an anatomical perspective. A linear mixed model analysis revealed that VA diameter was associated with sex, laterality (right or left), and an anomalous origin and abnormal foraminal entrance of the VAs. Additionally, the prevalence of an anomaly in either the V1 or V2 segment was 10% (24 of 238 patients), and the diameters of the anomalous VAs were considerably smaller (~2 mm) than those of normal VAs. These findings suggest that V1 and V2 anomalies may be useful marker for small or hypoplastic VA.

Currently, there is a lack of optimal criteria with which to accurately define VAH; there may be several reasons for this. First, VAH is considered to be a clinically benign condition and [21], therefore, digital subtraction angiography cannot be used as a gold standard method of assessment due to the invasiveness of this procedure [22]. Second, previous studies have used various criteria to define VAH in different populations [3–6, 8, 21, 23]. Importantly, as the cutoff value for VAH diameter and the age of the studied population increase, there is an escalating probability of a higher incidence of VAH. Additionally, the definition of hypoplasia includes the meaning ‘at birth’ and its presence should not change based on the definition of VA diameter, participants’ age, or the atherosclerotic burden of each patient. On the other hand, an anomalous origin of the VA and an abnormal entrance of the VA into the C6 TF are present at birth and do not change over time. Anomalous development of the VA is quite commonly accompanied by those of the adjacent bony structure such as an abnormal entrance of the vertebral artery not passing through the C6 TF [24–26]. Therefore, these characteristic anomalies of the VA and the TF could be consistent markers for defining small or hypoplastic VA.

During embryological development, the VA develops from the longitudinal anastomosis of the intersegmental arteries [11, 12]. An abnormal development of the caudal portion of the intersegmental artery leads to the formation of a VA with an aortic origin which, in turn, may lead to an increased risk of developing VAH compared to a person with a normal VA [27, 28]. In an autopsy study, the prevalence of VAs with an aortic origin was reported to be 2-6% [14, 29]. A similar prevalence of VAs with an aortic origin was identified in the present study using MRA. Likewise, VAH is associated with the development of spontaneous VA dissection [30] and the present findings show that VAH was also closely related with the abnormal development or congenital vasculopathy of the VA.

A previous study of the association between the VA and the TF revealed a close relationship between the diameter of the TF and VA flow/diameter [19, 25, 26, 31]. Additionally, Hong et al. [19] reported that the incidence of an abnormal penetration of the VA into the TF (other than through C6) is approximately 5% and that the diameter of the TF significantly differs whether the VA passes through it or not. The present observations are in line with these previous findings. Taken together, these findings indicate that there is a close relationship between the VA and the TF.

In the present study, a linear mixed model analysis was used to estimate the diameter of the VA. A mixed model is a hierarchical model used to examine the associations among between-subject and within-subject variables [32]. As a rule, there are two VAs in the body, and the left VA tends to be larger than the right one. Therefore, a hierarchical linear mixed model is an optimal method to account for the repeated measures (two VAs) used in the present study. Additionally, patients with atretic or non-visualized VAs on vessel images were often excluded from the analyses of previous studies, which can lead to an underestimation of the prevalence of VAH because smaller arteries are more easily narrowed or occluded than larger ones [3, 4, 6]. Although atretic or non-visualized arteries are considered as a missing value, which in turn excluded in a traditional linear regression analysis, the mixed model can take account these missing data into the final statistical model [33]. Therefore, when using a linear mixed model, the data from patients with atretic or non-visualized VAs can be included in the estimation of VA diameter. Thus, in conjunction with the constant radiological characteristics of the VA anomalies, the use of a mixed model to assess the diameters of VAs strengthened the present results.

However, the present study has several limitations that must be considered. First, this study included only patients who were examined using CT and MR angiography simultaneously and this inclusion criterion may have led to an unintended selection bias. However, the proportions of comorbidities in the anomalous and non-anomalous VA groups did not differ significantly, particularly for patients with atherosclerotic diseases such as ischemic stroke. Second, although it is reasonable to assume that young patients would be included in the present study population based on the definition of VAH and there was an effort to focus on young patients free of cerebrovascular disease, a large number of older participants (65.9 ± 12.0 years of age) were included in the present analyses. However, it is unreasonable to perform the CT angiography procedure on normal individuals to screen for VA diseases due to the hazards associated with the radiation and contrast agents.Finally, some stroke patients were included (*n* = 107subjects) in this study. Because they are not all patient with posterior circulation infarct, we could not conclude the relationship between the VA anomalies and the occurrence of ischemic stroke. In addition, there is a sample size issue because small number of VA anomalies were not sufficient for subgroup analysis. Therefore, it is necessary to study whether the presence of vertebral artery variation is associated with the occurrence of posterior circulation stroke.

## Conclusion

The present study demonstrated that the size of the VA was closely related with sex, laterality, and anomalies of the VA and that a linear mixed model was an optimal statistical method in assessing size of the VA.

## Additional file

Additional file 1: Are the anomalous vertebral arteries more hypoplastic. Raw data for the statistical analyses. Description of data: id, identification; gender; side (1: right, 2: left); V1, vertebral artery with an anomalous origin from the aorta (1: yes, 0: no); V2, vertebral artery not passing through the 6th cervical transverse foramen (1: yes, 0: no); Dia_VA, diameter of the vertebral artery (mm). (CSV 10 kb)

## Abbreviations

PCS: Posterior circulation stroke
TF: Transverse foramen
VA: Vertebral artery
VAH: Vertebral artery hypoplasia
